# SCYX-7158, an Orally-Active Benzoxaborole for the Treatment of Stage 2 Human African Trypanosomiasis

**DOI:** 10.1371/journal.pntd.0001151

**Published:** 2011-06-28

**Authors:** Robert T. Jacobs, Bakela Nare, Stephen A. Wring, Matthew D. Orr, Daitao Chen, Jessica M. Sligar, Matthew X. Jenks, Robert A. Noe, Tana S. Bowling, Luke T. Mercer, Cindy Rewerts, Eric Gaukel, Jennifer Owens, Robin Parham, Ryan Randolph, Beth Beaudet, Cyrus J. Bacchi, Nigel Yarlett, Jacob J. Plattner, Yvonne Freund, Charles Ding, Tsutomu Akama, Y.-K. Zhang, Reto Brun, Marcel Kaiser, Ivan Scandale, Robert Don

**Affiliations:** 1 SCYNEXIS, Inc., Research Triangle Park, North Carolina, United States of America; 2 Haskins Laboratory, Pace University, New York, New York, United States of America; 3 Anacor Pharmaceuticals, Inc., Palo Alto, California, United States of America; 4 Swiss Tropical and Public Health Institute, Basel, Switzerland; 5 Drugs for Neglected Diseases *initiative*, Geneva, Switzerland; Swiss Tropical and Public Health Institute, Switzerland

## Abstract

**Background:**

Human African trypanosomiasis (HAT) is an important public health problem in sub-Saharan Africa, affecting hundreds of thousands of individuals. An urgent need exists for the discovery and development of new, safe, and effective drugs to treat HAT, as existing therapies suffer from poor safety profiles, difficult treatment regimens, limited effectiveness, and a high cost of goods. We have discovered and optimized a novel class of small-molecule boron-containing compounds, benzoxaboroles, to identify SCYX-7158 as an effective, safe and orally active treatment for HAT.

**Methodology/Principal Findings:**

A drug discovery project employing integrated biological screening, medicinal chemistry and pharmacokinetic characterization identified SCYX-7158 as an optimized analog, as it is active *in vitro* against relevant strains of *Trypanosoma brucei*, including *T. b. rhodesiense* and *T. b. gambiense*, is efficacious in both stage 1 and stage 2 murine HAT models and has physicochemical and *in vitro* absorption, distribution, metabolism, elimination and toxicology (ADMET) properties consistent with the compound being orally available, metabolically stable and CNS permeable. In a murine stage 2 study, SCYX-7158 is effective orally at doses as low as 12.5 mg/kg (QD×7 days). *In vivo* pharmacokinetic characterization of SCYX-7158 demonstrates that the compound is highly bioavailable in rodents and non-human primates, has low intravenous plasma clearance and has a 24-h elimination half-life and a volume of distribution that indicate good tissue distribution. Most importantly, in rodents brain exposure of SCYX-7158 is high, with C_max_ >10 µg/mL and AUC_0–24 hr_ >100 µg*h/mL following a 25 mg/kg oral dose. Furthermore, SCYX-7158 readily distributes into cerebrospinal fluid to achieve therapeutically relevant concentrations in this compartment.

**Conclusions/Significance:**

The biological and pharmacokinetic properties of SCYX-7158 suggest that this compound will be efficacious and safe to treat stage 2 HAT. SCYX-7158 has been selected to enter preclinical studies, with expected progression to phase 1 clinical trials in 2011.

## Author Summary

Human African trypanosomiasis (HAT) is caused by infection with the parasite *Trypanosoma brucei* and is an important public health problem in sub-Saharan Africa. New, safe, and effective drugs are urgently needed to treat HAT, particularly stage 2 disease where the parasite infects the brain. Existing therapies for HAT have poor safety profiles, difficult treatment regimens, limited effectiveness, and a high cost of goods. Through an integrated drug discovery project, we have discovered and optimized a novel class of boron-containing small molecules, benzoxaboroles, to deliver SCYX-7158, an orally active preclinical drug candidate. SCYX-7158 cured mice infected with *T. brucei*, both in the blood and in the brain. Extensive pharmacokinetic characterization of SCYX-7158 in rodents and non-human primates supports the potential of this drug candidate for progression to IND-enabling studies in advance of clinical trials for stage 2 HAT.

## Introduction

Human African trypanosomiasis (HAT), commonly known as sleeping sickness, is a neglected disease caused by the kinetoplastid parasite *Trypanosoma brucei* and is fatal if left untreated.[1], [2] The parasite is transmitted through the bite of the tsetse fly, and is endemic in sub-Saharan Africa, where it is estimated that 50,000 people become infected every year.[3], [4], [5] The disease progresses through two distinct stages, an initial acute stage (stage 1) where the parasitic infection is restricted to the hemolymphatic system, and a second stage (stage 2) where the parasites have migrated across the blood-brain barrier and are resident in brain tissue.[6] This latter CNS stage is particularly difficult to treat, as the two drugs available for this purpose, melarsoprol and eflornithine, are toxic, have limited ability to cross the blood-brain barrier, and their activity is dependent upon complex parenteral administration procedures.[7], [8] Treatment failures are therefore quite common with these drugs. There are currently no orally active treatments for HAT. A quite interesting recent advance in clinical treatment of HAT has been the development of a treatment regime which employs nifurtimox and eflornithine in combination.[9], [10], [11], [12], [13] This combination still suffers from some of the drawbacks associated with the individual components (e.g. parenteral administration, toxicity, cost), but significantly reduces the complexity and duration of treatment in the clinical setting. Research efforts to discover new treatments for HAT have increased over the past several years, and have begun to deliver new biochemical targets and lead compounds.[14], [15]

**Figure 1:**
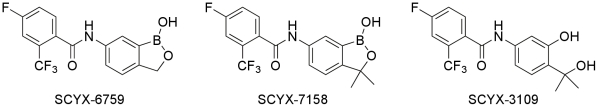
Chemical structures of compounds.

We have previously reported that screening of a library of benzoxaboroles from Anacor Pharmaceuticals (CA, USA) in a whole cell *T. brucei* viability assay revealed that these compounds are effective inhibitors of parasite growth at concentrations as low as 0.02 µg/mL.[16] From this initial screening effort, benzoxaborole 6-carboxamides were identified as attractive leads, as they exhibited good *in vitro* potency, activity in stage 1 mouse models of HAT and promising *in vitro* and *in vivo* pharmacokinetic properties.[17] This program yielded SCYX-6759 (see Figure 1 for chemical structures) - the first compound with sufficient potency, pharmacokinetic properties and blood-brain barrier permeability to provide complete cures in a stage 2 murine model of HAT. While SCYX-6759 was fully efficacious in the stage 2 mouse model following twice-daily intraperitoneal administration at a dose of 50 mg/kg (100 mg/kg/day) for 14 days, it exhibited only partial efficacy in the same model following twice-daily oral administration at 50 mg/kg for 7 days, and was not active at lower doses or when administered in a once-daily paradigm. This pharmacodynamic relationship was consistent with the *in vivo* pharmacokinetics of SCYX-6759 measured in mice, which demonstrated that, although this compound was well absorbed following oral administration and provided drug concentrations in plasma well in excess of the *in vitro* minimum inhibitory concentration (MIC), drug concentrations in the brain fell below the MIC by approximately 12 h post-dose following either single oral doses or a 7-day repeat oral dosing regimen matched to the stage 2 efficacy model. Consequently, further optimization of the lead series focused on improvement of the pharmacokinetic properties of the series, with particular emphasis on improving the extent and duration of brain exposure. Of several strategies pursued to improve brain exposure of the benzoxaborole 6-carboxamides, the approach which provided the best balance of potency and pharmacokinetics involved the installation of substituents at the C(3) position of the benzoxaborole scaffold. In particular, the C(3)-dimethyl analog SCYX-7158 (Figure 1) exhibited a profile supportive of progression to preclinical and clinical studies.

## Materials and Methods

### Ethics statement

Animal studies were carried out in accordance with the recommendations in the Guide for the Care and Use of Laboratory Animals of the National Institutes of Health. All pharmacokinetic (PK) studies were performed by AAALAC accredited facilities following an internal approval by their IACUC boards. Pharmacokinetic studies in rodents, beagle dogs and cynomolgus monkeys were performed by Vivisource Laboratories Inc (Waltham, MA; USDA# 14-R-0185, OLAW# A4543-01), Sinclair Research Center, LLC (Columbia, MO; USDA# 43-R-0122, OLAW# A4333-01) or SNBL USA (Everett, WA; USDA# 91-R-0053, OLAW# A4261-01), respectively. Blood collection from mice was performed under anesthesia; brain collection was performed following euthanasia. Collection of serial blood samples from rats was performed from tail vein venipuncture, or via a venous access port.

Efficacy studies were conducted in accordance with the recommendations in the Guide for the Care and Use of Laboratory Animals of the National Institutes of Health. The protocol was approved by the Institutional Animal Care and Use Committee of Pace University (Animal Assurance Welfare Number: A3112-01). Surviving animals are euthanized by carbon dioxide asphyxiation in sealed containers as approved by the American Veterinary Association. All efforts were made to minimize suffering; for example, in efficacy studies conducted at Pace University, animals were checked for parasitemia once per week and were immediately removed from cages and euthanized if parasites were found in a tail vein blood sample.

### Test compounds

SCYX-6759 [4-fluoro-*N*-(1-hydroxy-1,3-dihydrobenzo[c][1], [2]oxaborol-6-yl)-2-trifluoromethylbenzamide] and SCYX-7158 [4-fluoro-N-(1-hydroxy-3,3-dimethyl-1,3-dihydro-benzo[c][1], [2]oxaborol-6-yl-2-trifluoromethyl benzamide] were prepared as 10 mg/mL stocks in dimethyl sulfoxide (DMSO) for *in vitro* biological and absorption, distribution, metabolism and excretion (ADME) assays.

### Trypanosome and cell culture

The bloodstream-form *T. b. brucei* 427 strain was obtained from Dr. K. Stuart (Seattle Biomedical Research Institute, Seattle, WA) and was used for routine assessment of compound sensitivity *in vitro*. Additional strains used *in vitro* include *T. b. rhodesiense* STIB900, isolated in 1992 from a patient in Tanzania, and *T. b. gambiense* 108R, isolated from a patient in Democratic Republic of Congo in 2005. *T. b. brucei* EATRO 110 was a generous gift from the late William Trager of the Rockefeller University. *T. b. brucei* TREU 667 strains were kindly provided by F. W. Jennings at the University of Glasgow. Both *T. b. brucei* EATRO 110 and TREU 667 were cultured according to previously described conditions and used for *in vivo* studies.[18], [19]

*T. b. brucei* was cultured in complete HMI-9 medium,[20] which contains 10% fetal bovine serum (FBS) (Invitrogen, Carlsbad, CA), 10% Serum Plus medium (SAFC Biosciences, Lenexa, KS), and 100 units/mL penicillin and 0.1 mg/mL streptomycin. The trypanosomes were propagated in T-25 vented cap flasks (Corning Inc., Lowell, MA) at 37°C and 5% CO_2_ with humidity. To ensure log growth phase, trypanosomes were sub-cultured at appropriate dilutions (typically 1∶100) every 2–3 days in fresh HMI-9 medium. *T. b. rhodesiense* parasites were grown in MEM medium with Earle's salts supplemented with 15% horse serum. For *T. b. gambiense*, the MEM medium was supplemented with 5% heat-inactivated fetal calf serum (FCS) and 15% human serum.[21] L929 mouse fibroblast cells (ATCC CCL1, American Type Culture Collection, Rockville, MD) were used to determine parasite versus mammalian cell selectivity. Cells were cultured in Dulbecco's Modified Eagle Medium (DMEM), supplemented with 10% FBS, L-glutamine and 100 units/mL penicillin and 0.1 mg/mL streptomycin. MDCKII-hMDR1 cells were generously provided by P. Borst at the Netherlands Kancer Institute. Cells were cultured in DMEM with Glutamax, 10% (v/v) FBS, and 100 units/mL penicillin and 0.1 mg/mL streptomycin. Cell monolayers were fed with cell culture media 24 h after seeding and used for permeability studies 3 days later.

### *In vitro* compound sensitivity assays

Compounds to be tested were serially diluted in DMSO and added to 96-well plates to give final concentrations ranging from 5 to 0.01 µg/mL. *T. b. brucei* parasites in the log phase of growth were diluted in HMI-9 media and added to each well for a final concentration of 1×10^4^ parasites per well. For the sensitivity assays using *T. b. rhodesiense* and *T. b. gambiense*, pararasites were cultured in MEM supplemented with Baltz components (8), diluted in the aforementioned culture media, and added to each well at a density of 1×10^3^ cells/well. The final concentration of DMSO was 0.5% and the total volume was 100 µL/well. After a 72 h incubation, resazurin (Sigma-Aldrich, St. Louis, MO) was added to each well [20 µL of 25 mg/100 mL stock in phosphate buffered saline (PBS)] and incubated for an additional 4–6 h.[22] To assess cell viability, fluorescence was quantified using an EnVision Multilabel Plate Reader (Perkin Elmer, Waltham, MA) at an excitation wavelength of 530 nm and emission of 590 nm. Triplicate data points were averaged to generate sigmoidal dose-response curves and determine IC_50_ values using XLfit curve fitting software from IDBS (Guilford, UK). The IC_50_ is defined as the amount of compound required to decrease parasite or cell viability by 50% compared to those grown in the absence of the test compound. The MIC, defined as the lowest concentration of compound that completely inhibits visible parasite growth, was determined by visual inspection of 96-well plates after 48–72 h of incubation with the test compounds. To evaluate the effects of serum on trypanocidal activity, assays were performed in the presence of increasing concentration (2.5% to 50%) of fetal calf serum (Invitrogen, Carlsbad, CA). The results were expressed as a fold-change in IC_50_ values relative to standard conditions (10% FCS).

An evaluation of mammalian cell cytotoxicity was carried out in parallel with the trypanosome sensitivity assays. L929 mouse fibroblast cells were seeded at 2×10^3^ per well and handled as described above for the trypanosome sensitivity assay.

### Time-kill assays

The assessment of oxaborole-mediated killing of *T. b. brucei in vitro* was conducted using the CellTiter Glo kit (Promega, Inc., Madison, WI) to measure trypanosome ATP content as a real-time indicator of viability. Test compounds were serially diluted from 5 to 0.01 µg/mL into white wall-clear bottom 96-well plates (Corning Inc., Lowell, MA) containing HMI-9 media. 1×10^4^ trypanosomes were added to each well. At specified intervals, the CellTiter Glo reagent was added to lyse the parasites and the plates were incubated for 10 min in the dark. Luminescence was quantified using an EnVision plate reader. All determinations were done in duplicate. Time-kill parameters were determined from plots of parasite viability versus incubation time for each concentration tested.

### Examination of reversibility of trypanocidal effects

To establish the time and concentration required to cause persistent or irreversible effects by oxaboroles, *T. b. brucei* parasites were assessed for their ability to recover from transient exposure to test compounds. Trypanosomes were seeded in clear 96-well V-bottom plates at a density of 1×10^5^ parasites per well and incubated with serially diluted test compound (from 10 to 0.02 µg/mL). One plate was prepared for each time point. At the designated time, a plate was removed from the incubator and spun at 2,600 g for 5 min to sediment the parasites. The supernatant was aspirated and 100 µL of warmed HMI-9 media was added to each well. The wash was repeated twice more. The parasites were resuspended in 100 µL of warmed media and 20 µL of this suspension was added to 80 µL of HMI-9 media in triplicate plates. Following a 72 h incubation, resazurin was added and trypanocidal activity determined as described for the *in vitro* sensitivity assay.

### Efficacy in the mouse model for acute HAT

For efficacy studies against acute infections, groups of 3–5 female Swiss Webster mice (Ace Animals, Boyertown, PA) were injected intraperitoneally (i.p.) with freshly drawn infected rat blood containing 2.5×10^5^ trypanosomes (*T. b. brucei* EATRO 110 strain). The infection was allowed to progress for 24 h before treatment. Test compound was then given daily by bolus i.p. injection or oral gavage. Oxaborole compounds were formulated in 2% ethanol/5% dextrose and were given at a dose volume of 200 µL (containing 1.25 to 50 mg/kg) for a 25 g animal. Animals were monitored daily for signs of compound toxicity and clinical signs of trypanosomiasis for a period of 30 days. Mice were checked for parasitemia once per week by microscopic examination of smears prepared from the tail vein blood of animals. Animals remaining parasite free for more than 30 days beyond the end of the treatment period were considered cured. Control untreated animals typically succumbed to the infection within 4–5 days following i.p. inoculation with parasites.

### Efficacy in mouse model for chronic (CNS) HAT

For evaluation against late-stage CNS infections, a chronic disease inducing strain (TREU 667) of *T. b. brucei* was used.[19] Mice were divided into groups of ten and each animal was infected i.p. with 1×10^4^ parasites (200 µL). As a positive control, a group of mice were treated with a single 10 mg/kg dose of berenil (Sigma-Aldrich, St. Louis, MO) administered by i.p. injection on day 4 after infection. Infection in the remaining groups of animals was allowed to proceed for 21 days before treatment with either berenil (single i.p. dose at 10 mg/kg) or test compound given i.p. or orally twice daily for 7 or 14 days. Animals were checked for parasitemia once per week and were immediately removed from the cages and euthanized upon recrudescence. Animals were considered to be cured of a CNS infection if they were aparasitemic for at least 180 days after the end of the treatment period. Additionally, brain homogenates or blood of oxaborole-cured animals failed to generate infection when injected into mice immunosuppressed with cytoxan (40 mg/kg, QD x 2 d).

### Plasma protein and brain tissue binding

Binding to human or mouse plasma proteins or mouse brain homogenate was determined by rapid equilibrium dialysis (RED) (Pierce, Rockford, IL) using a 48-well plate-based format according to the manufacturer's instructions. Briefly, test compound at the required concentrations was added to fresh human or mouse plasma (Bioreclamation, Liverpool, NY) or freshly prepared mouse brain homogenate. Duplicate aliquots of each sample were transferred into the sample chambers of the RED devices, and dialysis buffer (BupH PBS) was added to the buffer chambers. The plates were sealed and incubated at 37°C for 4 h. After dialysis, samples collected from the buffer and tissue chambers were treated with ice-cold methanol (3 volumes for plasma, 4 volumes for brain) to precipitate proteins. The treated samples were centrifuged for 10 min at 3×10^3^ g at 15°C. The supernatants were assayed for test compound by LC-MS/MS. Calibration standards and quality control samples were prepared in matched matrix and assayed with samples. Values for unbound and bound fractions and mass balance were calculated. Concordance of binding for each batch of plasma was confirmed by assay of warfarin, imipramine and carbamezapine. Acceptance criterion for mass balance was 70–120%.

### *In vitro* metabolism

Metabolic stability was evaluated using mixed gender CD-1 mouse or human liver microsomal fractions (XenoTech, Lenexa, KS). Compounds (1 µM) were incubated with microsomes (1.05 mg/mL protein) for 0, 10, 15 and 30 min at 37°C in an oxygen and humidity enriched environment in the presence of an NADPH-regenerating system. Each compound was also incubated, under the same conditions, with CD-1 mouse, Sprague-Dawley rat, beagle dog, cynomolgus monkey, and human liver S9 sub-cellular fractions (2 mg/mL protein)[23] for 0, 15, 30 and 60 min. At the end of each incubation period, the reactions were quenched with 3 volumes of ice-cold methanol. Supernatants from the incubation mixtures were analyzed for parent compound by LC-MS/MS. Metabolic competencies of microsomal and S9 fractions were confirmed using control compounds 7-ethoxycoumarin, propranolol and verapamil. Intrinsic clearance (CL_int_) and half-life (t_½_) values were determined for each compound.

### *In vitro* prediction of blood-brain barrier permeability and P-glycoprotein (P-gp) mediated efflux transport

The propensity of the compound to cross the blood-brain barrier was examined using an *in vitro* MDCKII-hMDR1 transwell assay.[24] MDCKII-hMDR1 cells were seeded at density of 3×10^5^ cells per well onto microporous polycarbonate membranes in 12 well Costar Transwell plates (Corning Inc., Lowell, MA). The cells were used for permeability studies 3 days later. Trans-epithelial resistance (TEER) was measured for each insert to ensure the integrity of the monolayer (acceptable TEER >50 Ωcm^2^).

The permeability and propensity for P-gp-mediated efflux was evaluated by adding each compound at a concentration of 3 µM, in the presence or absence of 2 µM GF120918, to the apical compartment. Competency of the P-gp efflux transporter was confirmed by assay of propranolol (non-substrate) and amprenavir (substrate). Cell monolayers were incubated in triplicate with shaking (160 rpm) at 37°C in a 5% CO_2_-enriched humidified atmosphere for 1 h. Samples were removed from the apical and basolateral compartments after incubation and assayed for test compound concentrations by LC-MS/MS. Values for mass balance, P_app_A→B, P_app_A→B_+GF918,_ and absorption quotient (AQ) were calculated for each compound.[25], [26], [27] Acceptance criterion for mass balance was 70–120%.

### *In vitro* stability in mouse brain homogenate

The stability of each oxaborole in the CNS tissue was evaluated by incubation in fresh rodent brain homogenate. SCYX-7158 was incubated in freshly prepared homogenates of mouse brain tissue for 5 h at 37°C with gentle shaking. After incubation, the reactions were quenched with 4 volumes of ice-cold methanol. Supernatants from the incubation mixtures were analyzed for parent compound by LC-MS/MS.

### *In vitro* inhibition of cytochrome P450 enzymes

The potential for compound to exert drug-drug interactions that are mediated through inhibition of cytochrome P450 activities was assessed using P450-Glo assay kits (Promega Inc., Madison, WI). Assays for human cytochrome P450 isoforms 3A4, 1A2, 2C9, 2C19, and 2D6 were performed in triplicate according to the manufacturer's instructions over the concentration range 1–100 µM (n = 6 levels). Briefly, SCYX-7158 was added to membrane preparations containing human recombinant CYP450 enzymes together with luminogenic substrates specific to each isoform. Specific CYP450 inhibitors were included as positive controls (ketoconazole, CYP3A4; alpha-napthoflavone, 1A2; sulfaphenazole, CYP2C9; troglitazone, 2C19, and quinidine, CYP2D6). Reactions were initiated by addition of NADPH-regenerating solution and were allowed to incubate for an additional 15–30 min (isoform dependent). Luciferin detection reagent, containing the luciferin-labeled probe substrate, was then added. Luminescence was recorded on an EnVision Multilabel Plate Reader. Calculation of IC_50_ concentrations for each enzyme was determined using GraphPad Prism (V5.01). The potential for drug-drug interactions was characterized as high (IC_50_ <1 µM), moderate (1 – 10 µM), or low (IC_50_ >10 µM).[28]

### Pharmacokinetics studies in rodents, beagle dogs and non-human primates

The blood distribution, CNS disposition and pharmacokinetics of SCYX-7158 were evaluated in infected or non-infected rodents following intravenous or oral administration of compounds. Pharmacokinetics and bioavailability of SCYX-7158 were also evaluated in non-infected beagle dogs and cynomolgus monkeys. In-life phases of non-infected rodent studies were performed at Vivisource (Waltham, MA); studies in non-naïve cynomolgus monkeys were performed by SNBL USA (Everett, WA).

Male CD-1 mice (∼25 g), male Sprague-Dawley rats (∼225 g), or male cynomolgus monkeys (∼3–5 kg) were administered test article by either bolus intravenous injection (IV) or oral gavage. Animals in the IV groups received a single 2 mg/kg IV dose. Animals received oral doses of test articles as either single or twice daily doses ranging from 8 mg/kg to 50 mg/kg. All doses were administered as clear colorless solutions in either 50% (v/v) PEG 400: 20% (v/v) ethanol: 30% (v/v) carboxymethylcellulose (0.5% w/v in sterile water for injection) or 2% (v/v) ethanol: 5% (w/v) dextrose in sterile water for injection. Doses were administered in a volume of 4 mL/kg, 2 mL/kg or 1 mL/kg for mice, rats and cynomolgus monkeys, respectively. For pharmacokinetic analysis, blood samples were collected from mice via cardiac puncture under terminal anaesthesia. Serial blood samples were collected from rats via a vascular access port located in the lateral saphenous vein. Mice and rats were euthanized in a CO_2_ chamber before collection of terminal blood samples or brain tissue. Blood samples were collected into polypropylene tubes containing K_2_EDTA anticoagulant and stored on ice until centrifuged for the preparation of plasma. Plasma was stored at −70°C. Whole brains were collected following decapitation, blotted dry, placed into polypropylene containers and then immediately frozen at approximately −70°C. CSF was collected from rats via cisterna magna puncture, placed in sterile polypropylene tubes, and stored at approximately −70°C.

### Analysis of test compounds in biological samples

Samples of plasma (25 µL) and CSF (10 µL) were treated with 3 volumes of ice-cold methanol (containing 25 ng/mL of 2-chloro-4-fluoro-*N-*(1-hydroxy-1,3-dihyrobenzo[c][1], [2]oxaborol-6-yl)benzamide or 25 ng/mL d_6_-SCYX-7158 as an internal standard) to precipitate proteins. Treated samples were gently mixed at room temperature for 10 min, and then centrifuged at approximately 3000×*g* for 15 min at 15°C. The supernatants were transferred to 96-well plates or HPLC vials for analysis by LC-MS/MS.

Pharmacokinetic parameters were calculated from composite mean plasma or tissue data using non-compartmental (oral and intravenous routes) and bi-exponential (intravenous route) analyses in Microsoft Excel.

## Results and Discussion

### *In vitro* trypanocidal activity of SCYX-7158

In whole cell assays, SCYX-7158 exhibited potent activity against representative *T. b. brucei, T. b. rhodesiense* and *T. b. gambiense* strains. Parasite-mediated reduction of the pro-fluorescent dye resazurin was used as indicator for trypanosome viability. As shown in Table 1, IC_50_ values for SCYX-7158 were approximately 0.07 µg/mL to 0.37 µg/mL following incubation of the parasite strains with the compound for 72 h. In the *T. b. brucei* S427 strain, the MIC value for SCYX-7158 was 0.6 µg/mL, approximately two times the IC_50_ measured for this strain. In the *T. b. brucei* S427 assay, the primary oxidative metabolite SCYX-3109 (*vide infra*) was inactive, as it exhibited no inhibition of parasite growth at a concentration of 10 µM. In contrast to the potent activity of SCYX-7158 against trypanosomes, no significant inhibition of cell proliferation was observed in an *in vitro* mammalian cell (L929 mouse cell line) assay at drug concentrations up to 50 µg/mL.

**Table 1:** *In vitro* activity of SCYX-7158.

Parasite strain	IC_50_ (µg/mL)	Comments
*T. b. brucei* 427	0.292±0.019 (n = 9)	..
*T. b. rhodesiense* STIB 900	0.294 (n = 1)	Isolated from a patient in Tanzania in 1982, adapted to cell culture at Swiss Tropical Institute.
*T. b. gambiense* 108R	0.165 (n = 1)	Isolated from a patient in DRC in 2005, relapse 8 mo. after melarsoprol treatment.
*T. b. gambiense* 40R	0.363 (n = 1)	Isolated from a patient in DRC in 2005, relapse 6 mo. after melarsoprol treatment.
*T. b. gambiense* ITMAP 141267	0.092 (n = 1)	Isolated from a patient in DRC in 1960.
*T. b. gambiense* Drani	0.129 (n = 1)	Isolated from a patient in Uganda in 1995.
*T. b. gambiense* DAL 1402	0.065 (n = 1)	Isolated from a patient in Cote d'Ivoire in 1990
Mouse L929 fibroblast	> 50 (n = 4)	..

**Figure 2:**
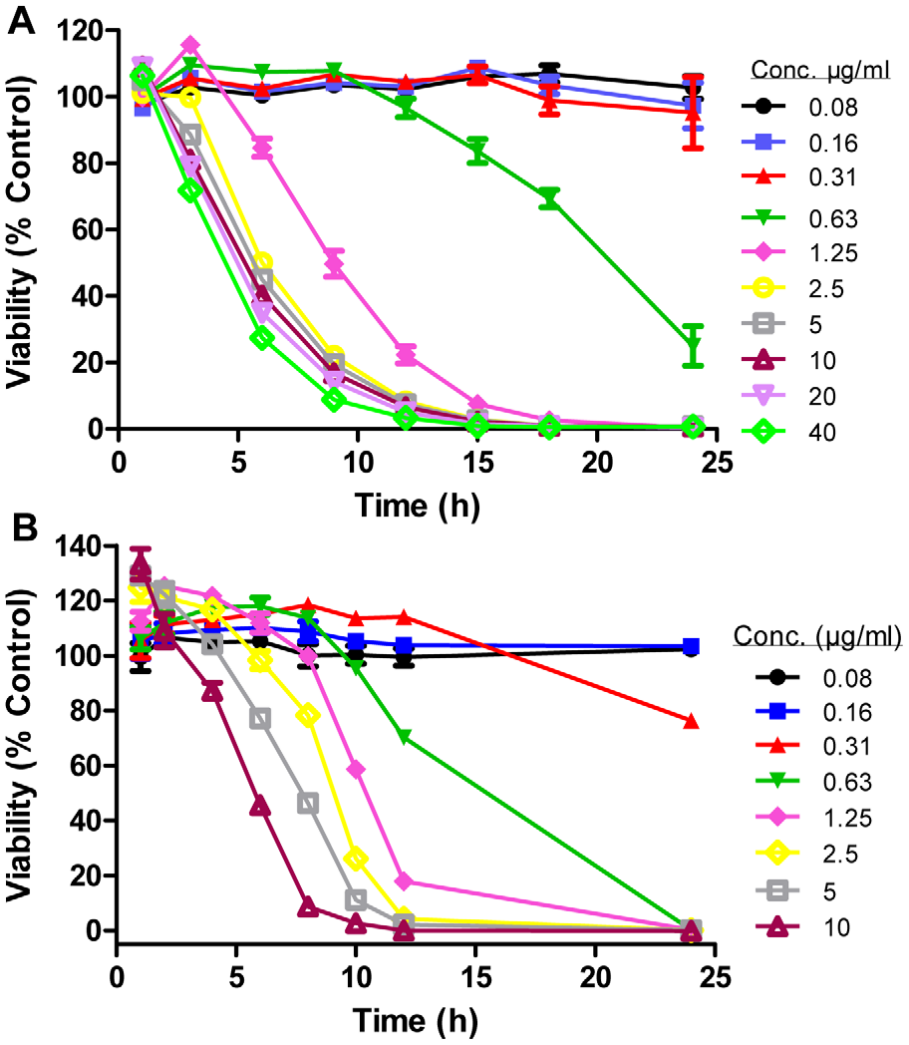
*In vitro* trypanocidal activity of SCYX-7158. **a**, Parasite viability, as indicated by ATP content, following continuous exposure of *T. b. brucei* 427 to SCYX-7158 at the indicated concentrations and times. Data are mean ± s.d. **b**, Irreversibility of trypanocidal effect. *T. b. brucei* 427 were exposed to the indicated concentrations of SCYX-7158 for the time indicated, then were sedimented by centrifugation and resuspended in drug-free media. Parasite viability was measured at 72 h by the resazurin method as described in the Methods section.

We next evaluated the *in vitro* time-kill relationship for SCYX-7158.[17], [29], [30] In these experiments, trypanosomes were exposed to continuous drug pressure and survival of the parasites was measured at several time points over 24 h. The results of this experiment, which measured parasite ATP content as an indicator of viability, are presented in Figure 2A. SCYX-7158 displayed concentration-dependent trypanocidal activity characterized by rapid onset, with greater than 50% reduction in viability within 8 h of exposure at a concentration (1.25 µg/mL) about 2 times the MIC (0.6 µg/mL). At these concentrations, >99% of the parasites were killed within 24 h of exposure to the compound.

The final aspect of activity of SCYX-7158 that was evaluated *in vitro* was the irreversibility of the trypanocidal effect. In these experiments (Figure 2B), we demonstrated that a short exposure (10–12 h) to the compound was sufficient to produce irreversible effects on trypanosome survival, albeit at a concentration of about 5 times the IC_50_. As was observed in the time-kill experiments, increasing the concentration of compound above this threshold value did not significantly alter the robustness or speed of its trypanocidal activity.

### *In vivo* trypanocidal activity of SCYX-7158

Our initial *in vivo* experiments were conducted in a mouse model of acute trypanosomiasis. In this model, mice were infected with 2.5×10^5^ parasites of the *T. b. brucei* EATRO 110 strain. This strain produces a robust infection in mice with rapid increases in parasitemia, ultimately leading to death of untreated mice within 4–5 days following infection.[18] Starting 24 h after infection, mice were administered an oral dose of SCYX-7158 once daily for 4 days. Mice treated with doses as low as 5 mg/kg/day (administered as either 5 mg/kg once daily (QD) or 2.5 mg/kg twice daily (BID)) exhibited a 100% cure rate (Table 2). Animals were monitored weekly for parasitemia through 30 days after infection. Those animals that remained parasite-free through the entire course of the experiment were considered cured.[18]

**Table 2:** Activity of SCYX-7158 in *T. b. brucei* acute mouse infections.

Dose (mg/kg)	Dose Frequency	Duration (days)	Route	Cured/Total	Avg. Days Parasite-Free	% Cured
10	BID	4	PO	5/5	>30	100%
10	QD	4	PO	5/5	>30	100%
5	BID	4	PO	5/5	>30	100%
5	QD	4	PO	5/5	>30	100%
2.5	BID	4	PO	5/5	>30	100%
2.5	QD	4	PO	0/5	8.4	0
1.25	BID	4	PO	0/5	8	0
1.25	QD	4	PO	0/5	4.4	0
25	QD	1	IP	3/3	>30	100%
10	QD	1	IP	0/3	9.3	0
5	QD	1	IP	0/3	6	0
25	QD	1	PO	1/3	8.3	33%

As the *in vitro* time-kill and irreversibility assays suggested that complete parasite clearance could be obtained within 12–24 h, we examined whether a single high dose of SCYX-7158 could produce a similar effect *in vivo*. In this experiment, a single 25 mg/kg i.p. dose of SCYX-7158 produced 100% cure by the criteria described above. Lower doses by the i.p. route, or oral administration of SCYX-7158 at 25 mg/kg, while not fully efficacious, increased the survival time of mice compared to untreated controls (Table 2).

The most important need for a clinically relevant HAT drug is the ability to cross the blood-brain barrier and kill parasites in the brain of the patient. In order to assess the potential of SCYX-7158 to address this need, a model of the stage 2 CNS HAT was employed.[31] In this model, mice are infected with 1×10^4^ parasites of the *T. b. brucei* TREU 667 strain, which produces a persistent hemolymphatic infection where parasites migrate across the blood-brain barrier within 21 days after infection.[19] Two control groups were included in each study – a positive control group in which animals were treated with berenil at day 4 after infection, and a negative control group in which animals were treated with berenil at day 21 after infection. Berenil does not cross the blood-brain barrier, so although it is very effective in clearing parasites from the hemolymphatic system, it is unable to clear parasites from the CNS. Consequently, animals treated on day 4 are cured as parasites have not yet crossed the blood-brain barrier; however, animals receiving berenil 21 days after infection do not sustain cures and recrudesce because parasites that have infected the CNS are able to migrate back across the blood-brain barrier and re-infect the hemolymphatic system generally around days 35–42 after infection.[32]

**Figure 3:**
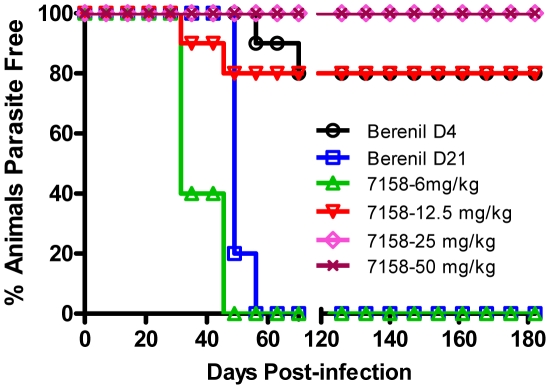
SCYX-7158 cures stage 2 trypanosomiasis in mice. Kaplan-Meier parasitemia plot for female Swiss-Webster mice (n = 10 per group) after infection with *T. b. brucei* TREU 667 (inoculum 1×10^4^ parasites). Oral treatment with SCYX-7158 started on day 21 after infection at the indicated doses (once daily for 7 days). Berenil (diminazene) was administered as a single 10 mg/kg dose intraperitoneally on either day 4 (positive control) or day 21 (negative control). Parasitemia was assessed weekly starting on day 21 by microscopic examination of a blood sample. Animals in which parasites were detected in the blood were sacrificed.

We examined SCYX-7158 at several doses in this model of Stage 2 HAT. When administered as a once daily oral dose of 12.5 mg/kg over 7 days starting on day 21 after infection, SCYX-7158 produced an 80% cure rate of the *T. b. brucei* infection, with a 100% cure rate observed following 7 daily oral doses of 25 mg/kg (Figure 3). In this model, cure is defined as lack of parasitemia in the blood for 180 days after the last dose, together with a lack of infection measured throughout 30 days in fresh animals inoculated with the blood and/or brain homogenates collected from the SCYX-7158 treated animals.

### *In vitro* ADMET characterization of SCYX-7158

Concurrent with our studies to explore the biological activity of SCYX-7158, we conducted extensive *in vitro* ADMET experiments to help understand the ability of this compound to be effective in the treatment of stage 2 HAT (Table 3). SCYX-7158 was metabolically stable when incubated with liver sub-cellular fractions from rodents, beagle dog, cynomolgus monkey and humans. Values for intrinsic clearance and half-life with all species were less than 5 µL/min/mg protein and longer than 350 min following incubation with microsomes and S9 fractions, respectively. When incubated with primary hepatocytes from rat or dog for extended periods (6–12 h), we observed the formation of two minor metabolites. Phase 1 oxidative deboronation of SCYX-7158 yielded the diol SCYX-3109 (Figure 1) as the primary metabolite; although less than 5% of the SCYX-7158 was metabolized over 12 h. Subsequent glucuronidation of SCYX-3109 was observed to a small degree. *In vitro* SCYX-7158 showed low clearance predictive of good exposure during in life studies.

**Table 3:** *In vitro* physicochemical and ADME properties of SCYX-7158.

Assay	Key Property	Value
Lipophilicity	logD	3.51
Aqueous Solubility	Solubility in pH 7.4 PBS	25 µM
Permeability – MDCK-MDR1 monolayer	P_app_ A→B	776 nm/sec
Permeability – MDCK-MDR1 monolayer	P_app_ A→B + GF120918	853 nm/sec
Permeability – MDCK-MDR1 monolayer	AQ	0.09
Inhibition of CYP1A2	IC_50_	>100 µM
Inhibition of CYP2C19	IC_50_	23.1 µM
Inhibition of CYP2C9	IC_50_	23.5 µM
Inhibition of CYP2D6	IC_50_	21.1 µM
Inhibition of CYP3A4	IC_50_	47.4 µM

The potential for SCYX-7158 to inhibit cytochrome P450 enzymes was evaluated using P450-Glo assays (Promega) for the human isoforms 3A4, 1A2, 2C19, 2C9 and 2D6. The IC_50_ values for SCYX-7158 in these assays were all above 10 µM, suggesting that this compound has low risk for CYP450-based drug-drug interactions.[28] We also evaluated the primary oxidative metabolite, SCYX-3109, in the CYP inhibition assays, where it exhibited IC_50_ values above 100 µM for the human isoforms 1A2, 2C19, 2C9 and 2D6. An IC_50_ of 7.5 µM for inhibition of human CYP 3A4 was measured.

The mechanism by which SCYX-7158 is trypanocidal is currently unknown. In order to assess potential biochemical targets (or target classes) that might be implicated in the mechanism of action of SCYX-7158, and to concurrently identify any potential off-target activities of SCYX-7158 that may hold potential for toxicity to the mammalian host, we evaluated SCYX-7158 in an array of *in vitro* receptor binding and enzyme inhibition assays. At a test concentration of 10 µM, SCYX-7158 did not exhibit any significant binding to, or inhibition of, any of the >100 biochemical targets tested. While these results did not identify the potential biochemical targets, we were encouraged because they predict a low risk of mechanism-based toxicity in our animal safety studies, which is also supported by the observation that SCYX-7158 was well tolerated by mice at doses up to 100 mg/kg BID (e.g. 200 mg/kg/day, 8 times the effective dose) which represents the highest dose administered during efficacy studies. This dose was based on practical considerations (e.g. solubility of compound in vehicle and dosing volume limitations). By way of comparison, in the closely related GVR35 stage 2 HAT model, melarsoprol must be dosed at 10–15 mg/kg QD × 5 days to effect cure, but is toxic to mice at 20 mg/kg QD x 5 days (R. Brun, personal communication). Preliminary toxicological evaluation of SCYX-7158 in rats and dogs is ongoing, and will be reported as part of a regulatory filing prior to initiation of clinical trials.

Due to implication of the hERG channel in potential cardiovascular toxicity, specifically long QT syndrome,[33] we evaluated SCYX-7158 in several hERG potassium channel assays. SCYX-7158 did not exhibit any significant binding to this channel in a radioligand binding assay (−6% at 10 µM), and we confirmed this result by investigation of the ability of SCYX-7158 to block the hERG channel expressed in HEK-293 cells using a whole-cell patch clamp technique.[34], [35] At test concentrations of 30 and 100 µM, SCYX-7158 produced a mean fractional block of 0.108±0.037 and 0.198±0.018, respectively, indicative of a hERG IC_50_ >100 µM.

The final *in vitro* toxicology study performed on SCYX-7158 was a bacterial reverse mutation (Ames) assay. In this assay, the potential for mutagenicity of SCYX-7158 was evaluated by exposure of several test strains of *Salmonella typhimurium* and *Escherichia coli*, both in the presence and absence of rat liver S9 fraction.[36] At a maximum dose of 5000 µg per plate, no positive mutagenic response was observed in any of the test strains employed, leading to the conclusion that SCYX-7158 is classified as an Ames-negative compound.

Binding of SCYX-7158 to human and mouse plasma proteins was determined by rapid equilibrium dialysis (RED, Pierce). Binding to plasma proteins was concentration dependent where the unbound fraction (fu) of SCYX-7158 in mouse plasma at the MIC (∼0.6 µg/mL) was 0.3% rising to 3.2% at plasma concentrations equivalent to C_max_ at steady-state (∼15 µg/mL, 25 mg/kg doses). Protein binding was modestly weaker in human plasma where unbound fraction was 1.3% and 5.5% with 1 µg/mL and 50 µg/mL SCYX-7158; the corresponding values for mouse plasma were 0.32% and 4.63%, respectively. Binding to mouse brain tissue was independent of concentration (fu_brain_ ∼5%). In these experiments, SCYX-7158 was also stable when incubated with plasma or brain homogenate for 4 h at 37°C.

To assess the possible impact of protein binding on potency, mouse or bovine serum was added to the *in vitro T. b. brucei* inhibition assay. The resulting *in vitro* IC_50_ was attenuated by less than 3–4 fold in the presence of 25% mouse serum or 50% bovine serum. These results suggest low affinity for protein binding (data not shown). In contrast, the *in vitro* potency of suramin, the current standard of care for stage 1 HAT (hemolymphatic stage), when tested under the same conditions, was attenuated by greater than 25 fold.

Successful treatment of stage 2 HAT requires therapeutically relevant exposure in the CNS compartment. To achieve this, SCYX-7158 needed to readily cross the blood-brain barrier and not be a substrate for the P-glycoprotein (P-gp) efflux transporter. An early prediction of the ability of SCYX-7158 to cross the blood-brain barrier was obtained by the assessment of the permeability in the MDCKII-hMDR1 monolayer transport assay.[24] In this assay, MDCKII cells over-expressing P-gp were grown to confluence on transwell membranes and incubated with SCYX-7158 (1.1 µg/mL). Apparent permeability in the apical to basolateral direction (P_app_A→B) was 776 nm/s, consistent with high blood-brain barrier permeability, where P_app_A→B values >150 nm/s are considered indicative of CNS exposure.[37] Furthermore, P_app_A→B values >50 nm/s predict rapid and complete absorption following oral administration.[26] When the permeability assay was performed in the presence of the known P-gp inhibitor GF120918, the P_app_A→B_+918_ value was similar at 853 nm/s. The absorption quotient (AQ), calculated from these two values (as described in the Methods section) was 0.09, indicating that SCYX-7158 is not a P-gp substrate.[25] Overall, the high P_app_A→B value and low AQ value predict that SCYX-7158 should readily cross the blood-brain barrier and, in light of good plasma pharmacokinetics, achieve therapeutically relevant exposures in brain tissue following oral dosing.

### *In vivo* pharmacokinetics of SCYX-7158

**Figure 4:**
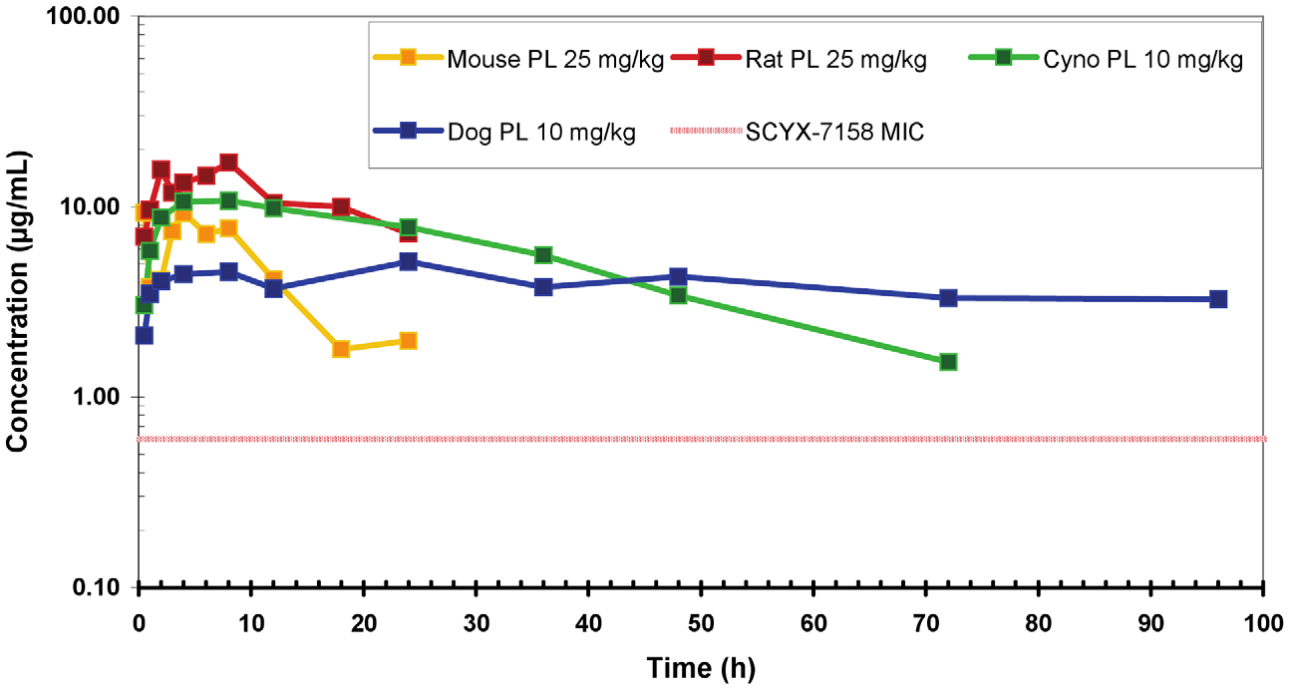
SCYX-7158 exhibits excellent plasma exposure across species. Male CD-1 mice, Sprague-Dawley rats, cynomolgus monkeys or male beagle dogs were administered a single oral dose of SCYX-7158 at a dose of 25 mg/kg (mouse, rat) or 10 mg/kg (monkey, dog). Blood samples were collected and analyzed as described in the Methods section. Data points for mouse and rat represent a single animal at each time point; data points for cynomolgus monkey and dog represent the mean of three animals at each time point. The MIC line (red hashed line) is defined as the lowest concentration of compound that completely inhibits visible parasite growth, determined by visual inspection of 96-well test plates after 72 h incubation.

Given the attractive *in vitro* ADME profile of SCYX-7158, we were confident that the compound would exhibit good *in vivo* pharmacokinetics. This expectation was confirmed in mice, rats, and monkeys following both intravenous and oral administration (Figure 4).

In uninfected mice, 4.3 mg/kg intravenous dose of SCYX-7158 showed an apparent elimination half-life (t_1/2_) of 26.6 h; systemic clearance (CL) of 0.089 L/h/kg; a volume of distribution (Vd_ss_) of 1.69 L/kg and area under the concentration-time curve (AUC_0–24 h_) of 48 h•µg/mL. Following an oral dose of 13.4 mg/kg, which corresponds to the lowest efficacious dose in the murine stage 2 HAT model, SCYX-7158 was rapidly absorbed, as a C_max_ of 6.96 µg/mL was achieved in plasma at 6 h after dose, with an oral clearance (Cl/F) value of 0.163 L/h/kg, an AUC_0–24 h_ of 82 h•µg/mL and absolute oral bioavailability of 55%. After a 26 mg/kg oral dose, which corresponds to the dose giving a 100% cure rate in the murine stage 2 HAT model, C_max_ increased to 9.8 µg/mL and the AUC_0–24 h_ was 113 h•µg/mL.

CNS exposure was determined in homogenates prepared from whole brain tissues. SCYX-7158 exhibited good permeability across the blood-brain barrier and achieved measurable levels after both intravenous and oral doses. Following intravenous administration at 4.3 mg/kg, SCYX-7158 demonstrated a brain AUC_0–24 h_ of 5.57 h•µg/mL. In the study where the compound was dosed orally at 13.4 mg/kg or 26 mg/kg, the values for C_max_ in brain tissue were 4.62 µg/mL and 8.1 µg/mL, respectively. The corresponding values for AUC_0–24 h_ were 31.4 h•µg/mL and 68 h•µg/mL. At these therapeutic doses, the brain to plasma ratios of SCYX-7158 were 38% and 60% based on AUC_0–24 h_ following the 13.4 mg/kg and 26 mg/kg oral doses, confirming that SCYX-7158 readily enters the CNS compartment and that disposition occurs in a dose-dependent manner.

In uninfected rats, following oral administration of SCYX-7158 at a nominal dose of 25 mg/kg (dose affording a 100% cure rate in mice), C_max_ increased approximately 2 fold more than that in mice (C_max_  = 18.2 µg/mL) and AUC_0–24 h_, and hence oral clearance, improved approximately 4 fold (AUC_0–24 h_ 291 h•µg/mL and CL/F  = 0.092 L/kg/h). The time to maximum concentration was similar to that in mice (t_max_  = 8 h).. In the same experiment, brain and cerebrospinal fluid (CSF) pharmacokinetics parameters were as follows: C_max_  = 8.5 µg/mL in the brain, 1.08 µg/mL in the CSF and AUC_0–24 h_  = 129 h•µg/mL in the brain and 14.6 h•µg/mL in the CSF. The brain to plasma AUC_0–24 h_ ratio was 44%, which is similar to that in mice.

The pharmacokinetic properties of SCYX-7158 were also evaluated in non-human primates following administration by intravenous and nasogastric (NG) routes. In this study, uninfected male and female cynomolgus monkeys were treated with SCYX-7158 at 2 mg/kg (IV) on study day 1 and 10 mg/kg (NG) on study day 8. Following each dose, blood samples were taken at 11 time points between 0.17 and 72 h, and CSF was collected at 2, 6, 12, 18 and 24 h in an off-set sparse sampling paradigm. As observed in both the mouse and rat studies, SCYX-7158 exhibited excellent plasma pharmacokinetics, with CL of 0.022 L/h/kg; Vd_ss_ of 0.656 L/kg and area under the concentration-time curve 78.8 h•µg/mL, and 94.4 for AUC_0–24 h_ and AUC_0–inf_, respectively, following intravenous administration. Pharmacokinetic properties were independent of gender. In the oral phase of the study, SCYX-7158 exhibited a C_max_ of 11 µg/mL at 9.5 h after dose, an oral clearance (Cl/F) value of 0.025 L/h/kg, an AUC_0–inf_ of 460 h•µg/mL, corresponding to absolute oral bioavailability of 89%. Concentrations of SCYX-7158 in the CSF of non-human primates were approximately 5% of the plasma concentration at each time point.

In this study, we also measured concentrations of the oxidative metabolite SCYX-3109 in plasma. In the 10 mg/kg NG group, the concentration of SCYX-3109 was approximately 1% of the SCYX-7158 concentration at all time points (e.g. C_max_  = 0.14 µg/mL and AUC_0-inf_  = 6.4 h•µg/mL). Though not measured due to technical limitations, we can estimate that the maximum concentration of boric acid (borate) would be no greater than 0.02 µg/mL, as it is generated on an equimolar basis to SCYX-3109. This concentration is below the background concentration (0.03 – 0.10 µg/g) reported for boric acid in the human population, and well below the concentration (1.27 µg/g) reported as a NOAEL in rats.[38], [39]

We have also measured plasma concentrations of the oxidative metabolite SCYX-3109 in separate studies in mice and rats. In mice, following a oral dose of 56 mg/kg SCYX-7158, the plasma concentrations SCYX-3109 were approximately 1% of the SCYX-7158 concentration at all time points, where the C_max_ for SCYX-7158 was 18 µg/mL and for SCYX-3109 was 0.27 µg/mL. Rats were administered an oral dose of 10 mg/kg of SCYX-7158, and concentrations of SCYX-3109 were 3-5% of the SCYX-7158 concentration at each time point. The C_max_ for SCYX-7158 was 14.3 µg/mL and for SCYX-3109 was 0.5 µg/mL. As in the non-human primate studies, boric acid concentrations were not measured in these studies, but the estimated maximum concentration could be calculated as 0.05 µg/mL and 0.09 µg/mL in mice and rats, respectively. As with the non-human primate studies, these concentrations of boric acid are well below the NOAEL in rats.[38], [39]

**Figure 5:**
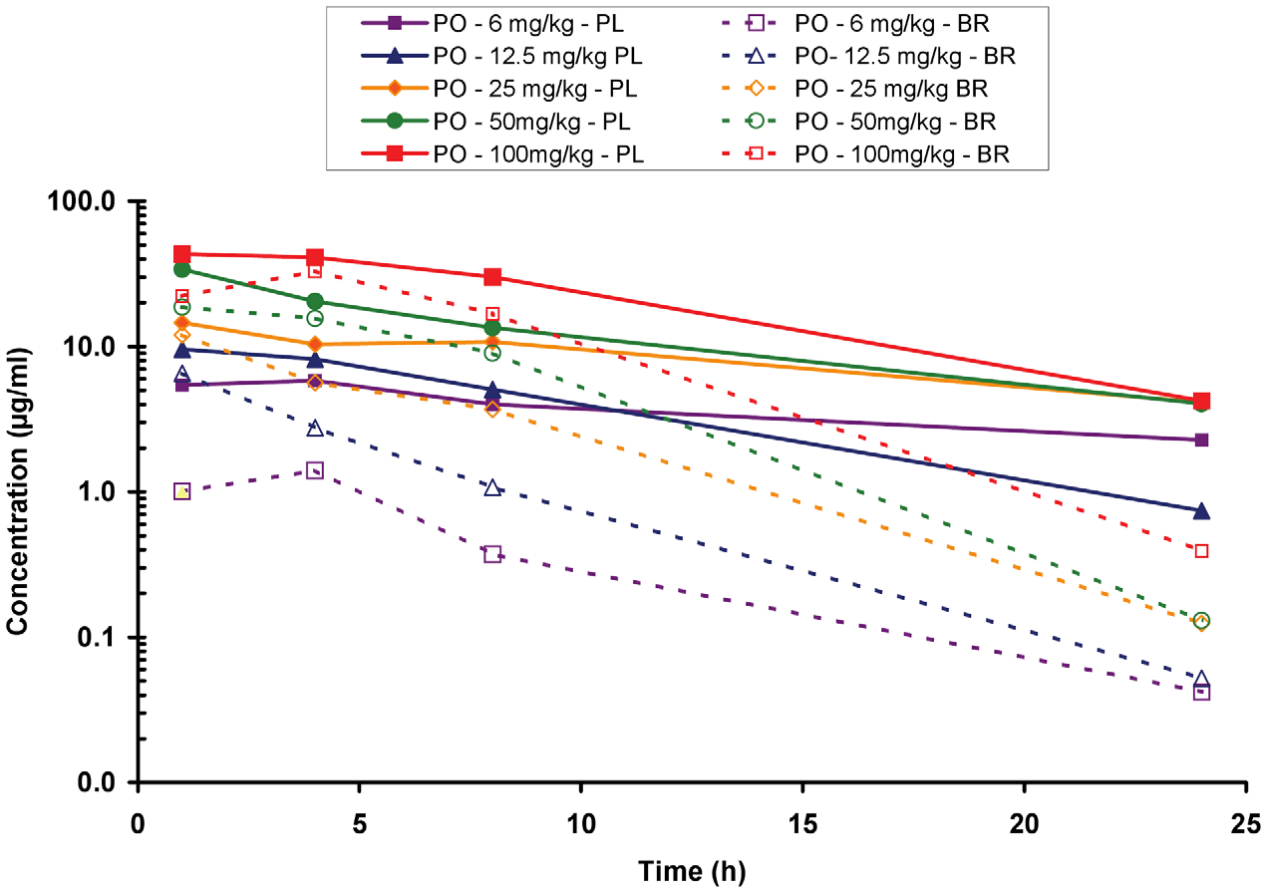
Time vs. concentration curves for SCYX-7158 following administration to mice infected with *T. b. brucei* TREU667. Female Swiss Webster mice were administered 7 daily doses of SCYX-7158 at the indicated doses. Blood (solid lines) and brain (dashed lines) samples were collected after the last dose and analyzed as described in the Methods section. Data points represent a single mouse at each time point. The MIC line (red hashed line) is defined as the lowest concentration of compound that completely inhibits visible parasite growth, determined by visual inspection of 96-well test plates after 72 h incubation.

Steady-state pharmacokinetic properties of SCYX-7158 were determined in *T. brucei*-infected mice after 7 daily oral doses of 6, 12.5, 25, 50 or 100 mg/kg. SCYX-7158 showed dose-related increases in exposure, although exposure increased in a less than proportional manner (Figure 5). Brain exposure was impressive in these mice as well. For example, following a 12.5 mg/kg oral dose, a C_max_ of 6.54 µg/mL and an AUC_0-inf_ of 34.2 h•µg/mL were achieved in mouse brain tissue; at 25 mg/kg, C_max_ of 11.98 µg/mL and an AUC_0-inf_ of 81.5 h•µg/mL were observed. Most importantly, brain concentrations of SCYX-7158 were maintained at or above the MIC (0.6 µg/mL) for close to 24 h following the 25 mg/kg dose, consistent with the efficacy observed in the stage 2 HAT assay. Interestingly, efficacy correlated with total brain exposure rather than the unbound concentration. Consistent with minimal attenuation of in vitro potency in the presence of serum, this likely reflects weak, non-restrictive, binding of SCYX-7158 to brain tissue complemented with rapid distribution into trypanosomes.

The pharmacokinetic properties and efficacy of SCYX-7158 in the stage 2 murine HAT model have demonstrated that a once-daily oral regimen is possible during clinical application. Efficacy in the stage 2 model was dependent on maintaining brain concentrations above the MIC for approximately 24 h or achieving brain concentrations above 3 times the MIC for shorter periods of time, as depicted in Figures 2B and 5. If pharmacokinetic clearance scales from pre-clinical species to humans based on body weight or hepatic blood flow are likely, clinical dose would be approximately 2.5 mg/kg, although this must await confirmation in clinical trials. These observations along with *in vivo* pharmacodynamic relations observed in the mouse stage 2 HAT experiments, where maintenance of drug concentration at or above the trypanocidal MIC for 14–20 h was sufficient to demonstrate cures in this model, suggest that SCYX-7158 could be a once-daily oral treatment for HAT.

### Concluding Remarks

The optimization of a series of benzoxaboroles discovered to exhibit parasitical activity against *T. brucei* has culminated in the identification of SCYX-7158. We have demonstrated that this compound is potent in *in vitro* trypanocidal assays and has attractive *in vitro* physicochemical and ADME properties. In animal models of HAT, SCYX-7158 exhibits significant activity following oral administration, including cure of a CNS *T. brucei* infection following 7 days administration at a dose of 25 mg/kg. The *in vivo* pharmacokinetic characterization of SCYX-7158 reveals that this compound is highly bioavailable across species, and can cross the blood-brain barrier to achieve therapeutically-relevant concentrations in the brain and cerebrospinal fluid of rodents. Based on these properties, SCYX-7158 has been selected to enter IND-enabling preclinical studies, with expected progression to phase 1 clinical trials in 2011.
